# The role of T2*-weighted gradient echo in the diagnosis of tumefactive intrahepatic extramedullary hematopoiesis in myelodysplastic syndrome and diffuse hepatic iron overload: a case report and review of the literature

**DOI:** 10.1186/s13256-017-1531-9

**Published:** 2018-01-15

**Authors:** Abel A. Belay, Andrew M. Bellizzi, Alan H. Stolpen

**Affiliations:** 1 Department of Diagnostic Radiology, University of Iowa Hospitals and Clinics, 200 Hawkins Dr, Iowa City, IA 52242 USA; 2 Department of Pathology, University of Iowa Hospitals and Clinics, 200 Hawkins Dr, Iowa City, IA 52242 USA

**Keywords:** Myelodysplastic syndrome, Iron, MRI, Hepatic extramedullary hematopoiesis, T2*

## Abstract

**Background:**

Extramedullary hematopoiesis is the proliferation of hematopoietic cells outside bone marrow secondary to marrow hematopoiesis failure. Extramedullary hematopoiesis rarely presents as a mass-forming hepatic lesion; in this case, imaging-based differentiation from primary and metastatic hepatic neoplasms is difficult, often leading to biopsy for definitive diagnosis. We report a case of tumefactive hepatic extramedullary hematopoiesis in the setting of myelodysplastic syndrome with concurrent hepatic iron overload, and the role of T2*-weighted gradient-echo magnetic resonance imaging in differentiating extramedullary hematopoiesis from primary and metastatic hepatic lesions. To the best of our knowledge, T2*-weighted gradient-echo evaluation of extramedullary hematopoiesis in the setting of diffuse hepatic hemochromatosis has not been previously described.

**Case presentation:**

A 52-year-old white man with myelodysplastic syndrome and marrow fibrosis was found to have a 4 cm hepatic lesion on ultrasound during workup for bone marrow transplantation. Magnetic resonance imaging revealed diffuse hepatic iron overload and non-visualization of the lesion on T2* gradient-echo sequence suggesting the presence of iron deposition within the lesion similar to that in background hepatic parenchyma. Subsequent ultrasound-guided biopsy of the lesion revealed extramedullary hematopoiesis. Six months later, while still being evaluated for bone marrow transplant, our patient was found to have poor pulmonary function tests. Follow-up computed tomography angiogram showed a mass within his right main pulmonary artery. Bronchoscopic biopsy of this mass once again revealed extramedullary hematopoiesis. He received radiation therapy to his chest. However, 2 weeks later, he developed mediastinal hematoma and died shortly afterward, secondary to respiratory arrest.

**Conclusions:**

Mass-forming extramedullary hematopoiesis is rare; however, our report emphasizes that it needs to be considered in the initial differential diagnosis of hepatic lesions arising in the setting of bone marrow disorders. We also show that in the setting of diffuse hepatic iron overload, tumefactive extramedullary hematopoiesis appeared isointense to background liver on T2* gradient-echo sequence, while adenoma, hepatoma, and hepatic metastasis appear hyperintense. Thus, T2*-weighted gradient-echo sequence may have a potential role in the imaging diagnosis of mass-forming hepatic extramedullary hematopoiesis arising in the setting of diffuse iron overload.

## Background

In adults, extramedullary hematopoiesis (EMH) is seen in the setting of marrow-occupying lesions like myelofibrosis, myeloproliferative disorders, and carcinoma with extensive bone marrow metastasis and in the setting of increased cell turnover (for example, chronic hemolytic anemia) [1–3]. The most common sites of EMH in the abdomen are the liver and spleen; and when involving the liver and spleen, EMH most commonly presents as hepatosplenomegaly [2]. With many underlying causes of EMH, patients require multiple blood transfusions, which frequently result in diffuse hepatosplenic hemochromatosis. In one study, the prevalence of hepatic iron overload in those with transfusion-dependent anemias in Europe was reported to be approximately 51% [4]. EMH rarely presents as a focal mass with involvement of the liver and spleen [2]. In such cases, differentiation from primary and metastatic hepatic lesions based on imaging can be difficult and a biopsy is often performed for definitive diagnosis. We report a case of tumefactive hepatic EMH in the setting of myelodysplastic syndrome (MDS) with concurrent hepatic iron overload, and the role of T2*-weighted gradient-echo (GRE) magnetic resonance imaging (MRI) in differentiating EMH from primary and metastatic hepatic lesions. To the best of our knowledge, T2*-weighted GRE evaluation of EMH in the setting of diffuse hepatic iron overload has not been previously described.

## Case presentation

A 52-year-old white man with chronic obstructive pulmonary disease, 15 pack-years of tobacco smoking history, moderate use of alcohol, and no significant family history presented with pre-syncopal episode and was found to be pancytopenic. His vital signs were temperature 36.7 °C, pulse 93 beats/minute, respiration rate 20 breaths/minute, blood pressure 101/60 mmHg, and blood oxygen saturation (SpO_2_) 99% at room air. A physical examination including neurological examination was unremarkable. Comprehensive metabolic profile and urine analysis was normal. Complete blood count showed pancytopenia. A peripheral blood smear showed a myeloid left-shift with 5% blasts. Subsequent bone marrow biopsy showed erythroid dyspoiesis, left-shifted granulopoiesis, and grade 3+ fibrosis consistent with MDS with marrow fibrosis. Genetic testing revealed wild-type *JAK2* and *MPL*, a normal male karyotype, lack of BCR-ABL1 translocation, and a negative MDS fluorescence *in situ* hybridization (FISH) panel. Based on the lack of cytogenic abnormalities, hemoglobin (Hgb) of 7.8 g/dL, platelets (Plt) of 32 × 10^9^/L, absolute neutrophil count (ANC) of 11.3 × 10^9^/L, and the presence of 5% blasts, an age-adjusted Revised International Prognostic Scoring System (IPSS-R) score of 5.1 was calculated (that is, high risk – with a median survival and time to progression to acute leukemia of 1.6 and 1.4 years, respectively) [5]. He received multiple blood transfusions for pancytopenia and was treated with 5 mg twice a day of the Janus kinase inhibitor, ruxolitinib.

**Fig. 1:**
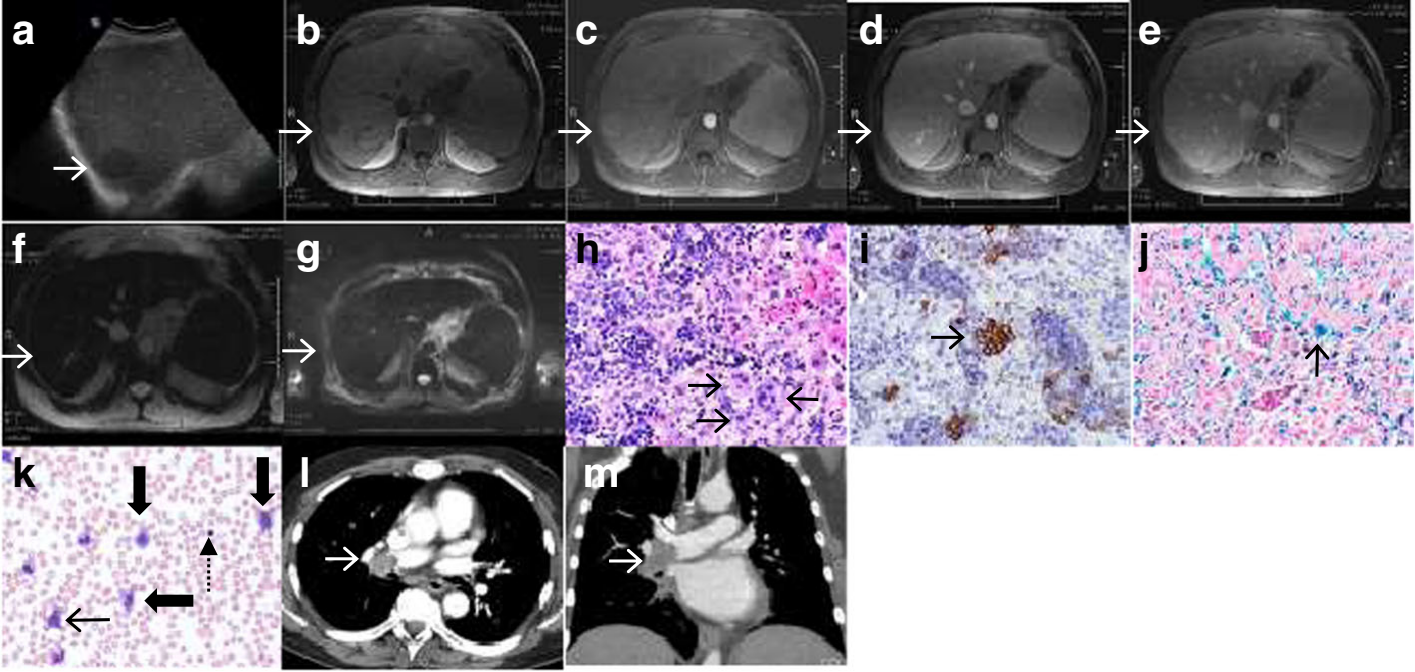
**a** Liver sagittal ultrasound shows a hypoechoic lesion in the right hepatic lobe (*white arrow*). **b** Axial T1-weighted volumetric interpolated breath-hold examination pre-contrast image shows a lesion in segment 7 (*white arrow*) that is hypointense to hepatic parenchyma. **c** After 20 cc of intravenous MultiHance® contrast agent administration heterogeneous mild enhancement was detectable in the arterial phase (*white arrow*). **d**, **e** The lesion washes out to isointensity in the portal venous and 5-minute delayed phases. **f** Axial T2*-weighted gradient echo shows decreased liver and spleen signal intensity, lower than that of the paraspinal musculature, consistent with diffuse parenchymal iron deposition, with non-visualization of the segment 7 lesion due to iron deposition similar to the rest of the hepatic parenchyma (*white arrow*). **g** Diffusion-weighted axial scan shows absence of restriction and non-visualization of the lesion (*white arrow*). **h** Hematoxylin and eosin stain demonstrates a sinusoidal-based infiltrate of bone marrow elements, including three megakaryocytes at the lower right (*black arrows*). **i** C-KIT immunostain highlights frequent myeloid blasts (*black arrow*), reflecting this patient’s evolving myelodysplastic syndrome. **j** Perls’ iron stain highlights massive iron accumulation (*black arrow*). **k** Blood smear with Wright’s stain, × 500, oil emersion demonstrates a peripheral monocytosis (the larger cells with convoluted nuclei and pale, basophilic, vacuolated cytoplasm, *thick arrows*) and left-shifted granulopoiesis with a few bands and a single myeloid blast (*thin arrow*); a nucleated red blood cell is also noted in the right half of the image (*dashed arrow*). **l**, **m** Computed tomography angiogram of the chest shows an enhancing mass within the right main pulmonary artery suggestive of tumor thrombus (*white arrow*)

Three months after diagnosis of MDS with fibrosis, during workup for allogenic bone marrow transplantation (BMT), a 4 cm right hepatic lesion was discovered on ultrasound. The lesion was hypoechoic (Fig. 1a) with internal vascularity. Hepatosplenomegaly was also noted with the spleen measuring 19 cm in the craniocaudal dimension. MRI performed with MultiHance® revealed diffuse hepatic iron deposition and a T1 hypointense (Fig. 1b) and T2 isointense (not shown) lesion in segment 7 showing heterogeneous mild arterial phase enhancement (Fig. 1c) with washout to isointensity on portal venous (Fig. 1d) and delayed phase images (Fig. 1e). Of note, the lesion was not visible on T2*-weighted GRE (Fig. f) images suggesting the presence of iron deposition within the lesion similar to that in background hepatic parenchyma. The lesion was also not visible on diffusion-weighted imaging (DWI; Fig. 1g). The differential diagnosis offered at this point included focal nodular hyperplasia (FNH) and adenoma, with hepatocellular carcinoma (HCC) being less likely and metastasis being unlikely. A few days later, our patient underwent ultrasound-guided core needle biopsy of the lesion, revealing marked sinusoidal EMH (Fig. 1h) with left-shifted granulopoiesis including increased blasts, the latter highlighted on c-KIT immunostain (Fig. 1i). Perls’ iron stain highlighted 4+ iron in hepatocytes and Kupffer cells (Fig. 1j).

After approximately 7 months of therapy with ruxolitinib, a repeat bone marrow biopsy did not show any change in the blast count but a peripheral smear showed 7% blasts, meeting criteria for refractory anemia with excess blasts-2 (RAEB-2) using the 2008 World Health Organization (WHO) classification system for MDS subtypes [6]. Two weeks later, while still being evaluated for BMT, pulmonary function tests revealed a poor forced expiratory volume at 1 second (FEV1) of 60% and a disproportionately reduced diffusing capacity of the lung for carbon monoxide (DLCO) of 56% predicted. This prompted a computed tomography (CT) angiogram of his chest, which showed an enhancing mass within his right main pulmonary artery suggestive of tumor thrombus (Fig. 1l and m). Bronchoscopic aspiration of the mass also showed EMH with dyspoiesis. He shortly developed hemoptysis at which point treatment with ruxolitinib was stopped and he was treated with low-dose radiotherapy to his chest (1 to 2 Gy/5 to 10 fractions) for approximately 10 days. A third bone marrow examination performed shortly afterwards showed similar findings to the previous with the additional finding of a significant peripheral monocytosis, at which point the disease was best classified as chronic myelomonocytic leukemia-2 (CMML-2) (Fig. 1k). Two weeks after diagnosis of CMML-2 and 3 weeks after the CT angiogram, he developed mediastinal hematoma probably due to progressive CMML-2 and radiation-induced degeneration of the pulmonary arterial EMH; he died shortly afterward, secondary to expanding mediastinal hematoma causing respiratory arrest. An autopsy was not performed to the best of our knowledge.

## Discussion

EMH rarely presents as a focal mass in the liver and spleen in the context of various bone marrow disorders. In our patient with MDS, the initial differential diagnoses we offered after performing an ultrasound and MRI of the hepatic lesion were FNH and adenoma with HCC being less likely and metastasis being unlikely. The diagnosis of EMH was only made after biopsy. Our case report shows that, despite its rarity, mass-forming EMH should be included in the differential diagnosis whenever patients with bone marrow disorders present with a focal hepatic mass. Furthermore, we show that the mass-forming hepatic EMH arising in the setting of secondary diffuse hepatic hemochromatosis (a common presentation in the context of bone marrow disorders due to multiple blood transfusions) was isointense to background liver on T2*-weighted GRE sequence indicating that the lesion contained as much iron as the background hepatic parenchyma. On the other hand, primary and metastatic hepatic lesions are known to usually appear hyperintense on T2* GRE sequence in such settings, indicating they exclude iron. Our case report thus provides the first description (to the best of our knowledge) of the T2*-weighted GRE characteristics of EMH in the setting of diffuse hepatic iron overload and the potential use of T2*-weighted GRE sequence in differentiating EMH from primary and metastatic hepatic lesions.

Some studies have reported the T2*-weighted GRE characteristics of hepatic metastasis and hepatoma (HCC) after administration of iron oxide contrast agents. Namasivayam *et al.* have shown that iron oxide particles such as ferumoxides are taken up by functioning Kupffer cells, while metastases do not contain Kupffer cells, and thus lack the capacity to phagocytose iron oxide particles [7]. Hence, the MR signal of liver metastases remains unchanged on T2*-weighted GRE images after intravenously administered injection of ferumoxides, whereas normal liver shows marked signal loss due to T2* relaxation caused by the strong local field inhomogeneities created by the iron oxide agents. In addition, Zhang and Krinsky [8] and Kashala *et al.* [9] have shown that HCCs, including well-differentiated ones, tend to lose the ability to accumulate iron, and when they arise in the setting of iron overload, they are often iron deficient appearing hyperintense on T2*-weighted images against a dark liver parenchyma.

Grazioli *et al*. show that adenomas and FNH can show variable uptake of iron oxide-based contrast agents based on Kupffer cell content and function [10, 11]. Adenomas have either absent or few non-functioning Kupffer cells [12]. As a result, they show usually poorer or heterogeneous superparamagnetic iron oxide (SPIO) uptake compared to that occurring in the surrounding normal liver parenchyma leading to hyperintense signal on post-contrast T2*-weighted GRE images [10]. Unlike adenomas, FNHs contain functional Kupffer cells in variable amounts and show SPIO uptake to a much greater extent compared to adenomas. Some studies have shown that FNH can show 70 to 100% signal drop out after SPIO administration on T2-weighted turbo spin echo (TSE) sequences [11, 13]. However, to the best of our knowledge, there is no literature describing T2*-weighted GRE characteristics of FNH after iron oxide contrast agent administration.

Tumefactive EMH, on the other hand, should show a signal drop out on T2*-weighted GRE images comparable to background hepatic parenchyma when iron oxide-based contrast agents are used. This is because EMH possesses the full array of marrow cells including cells of the reticuloendothelial system (RES) where marrow iron is physiologically stored. Macrophages in EMH would thus phagocytose iron compounds to a similar extent as hepatic Kupffer cells and would remain indistinguishable from background liver parenchyma on T2*-weighted GRE images [14]. While this is often the case, exceptions have been reported, presumably due to variable affinities of iron-based contrast agents to different cells. For instance, Resovist® (SHU 555A) has the strongest affinity to Kupffer cells, and the least affinity to macrophages in bone marrow. At least one instance of a weak or even no signal loss of intrahepatic EMH has been reported which was thought to be in part due to the low affinity of Resovist® to marrow macrophages [1].

In our case, iron-based contrast agents were not given (we used MultiHance); however, diffuse hepatic iron overload was already present secondary to multiple blood transfusions. In this setting, the tumefactive EMH showed signal loss comparable to the iron-overloaded hepatic parenchyma appearing isointense to background liver on T2*-weighted GRE sequence. This makes intuitive sense because in diffuse secondary hemochromatosis excess iron is deposited in the cells of RES mimicking uptake characteristics of iron-based contrast agents. Another possible reason for the comparable loss of signal in EMH and iron-overloaded hepatic parenchyma is the relationship of EMH to the hepatic sinusoids. In animals, when tumefactive hematolymphoid lesions including EMH involve the liver, they may present as one of three possibilities: sinusoidal-based lesions (most common), portal-based lesions, or mass-forming lesions excluding background liver [15]. In our case, the EMH was both sinusoidal based and mass forming excluding background liver. A given cross-sectional area of the latter type of EMH consists of approximately half EMH and half background liver (Fig. 1h). Uptake of iron by Kupffer cells in the liver tissue admixed with the tumefactive EMH would thus have contributed to at least part of the signal loss on T2*-weighted GRE sequences.

Similar to iron oxide-based agents, in the setting of diffuse iron overload, HCC and hepatic metastasis would be expected to stand out as hyperintense lesions against the hepatic parenchyma on T2*-weighted GRE images; and FNH and adenoma would be expected to show variable level of uptake based on Kupffer cell content and function.

When diffuse hepatic hemochromatosis is present and is used as “internal contrast,” a better tumor-to-liver contrast may be achieved as it is not subject to the same limitations of iron oxide-based contrast agents. For instance, affinity-related limitations of iron contrast agents may not apply in the setting of diffuse secondary hemochromatosis. Gale *et al*. have shown that in a chemical analysis of iron concentration in the marrows and livers of 199 necropsy individuals, the liver and bone marrow show very similar concentrations of iron in individuals with depleted stores, normal stores, and all degrees of iron overload [16]. In other words, EMH containing marrow macrophages and hepatic Kupffer cells should show similar iron uptake over a wide range of iron concentrations leading to a comparable T2* signal loss.

We recognize, however, that other forms of hepatic iron overload exist, including focal siderosis, hypersiderosis, periportal siderosis, and diffuse siderosis with focal areas of hepatic iron sparing [17]. Our discussion is not applicable to the evaluation of EMH in these types of hepatic iron overload, and the T2*-weighted GRE characteristics of EMH in these settings remain to be determined. In addition, the role of T2*-weighted GRE sequence in the evaluation of tumefactive EMH in the non-iron-overloaded liver is not known. It has been reported that newly formed, active EMH lesions have rich vascularity, whereas older “burned out” lesions have massive iron deposition and fat infiltration [18], suggesting a potential role in the T2* GRE evaluation of at least older EMHs arising in the non-iron-overloaded liver.

## Conclusions

Mass-forming EMH is rare, however, our case report emphasizes that it needs to be considered in the initial differential diagnosis of hepatic lesions arising in the setting of bone marrow disorders. We also show that in the setting of diffuse hepatic iron overload, tumefactive EMH did not exclude iron and appeared isointense to background hepatic parenchyma on T2* GRE sequence, in contrast to adenoma, HCC, and hepatic metastasis which exclude iron and usually appear hyperintense. Thus, T2*-weighted GRE sequence may have a potential role in the imaging-based diagnosis of mass-forming EMH at least in the setting of diffuse secondary hepatic hemochromatosis. When imaging is not pathognomonic, biopsy remains the reference standard for definitive diagnosis.
